# Formulation and Drug Loading Features of Nano-Erythrocytes

**DOI:** 10.1186/s11671-017-1980-5

**Published:** 2017-03-17

**Authors:** Xiaoting Dong, Yawei Niu, Yi Ding, Yuemin Wang, Jialan Zhao, Wei Leng, Linghao Qin

**Affiliations:** 1 Department of Pharmaceutics, School of Pharmacy, Guangdong Pharmaceutical University, No. 280 Waihuandong Road, Guangzhou, 510006 China; 2 Guangzhou Institute for Drug Control, 23 Xizeng Road, Guangzhou, 510160 China; 3 Jacobson Pharma Group, 7 Dai Shun Street, Tai Po District, NT Hong Kong

**Keywords:** STS, Erythrocyte ghosts, Drug delivery, Repairing effect

## Abstract

Nano erythrocyte ghosts have recently been used as drug carriers of water-soluble APIs due to inherit biological characteristics of good compatibility, low toxicity, and small side-effect. In this study, we developed a novel drug delivery system based on nano erythrocyte ghosts (STS-Nano-RBCs) to transport Sodium Tanshinone IIA sulfonate (STS) for intravenous use in rat. STS-Nano-RBCs were prepared by hypotonic lysis and by extrusion methods, and its biological properties were investigated compared with STS injection. The results revealed that STS-Nano-RBCs have narrow particle size distribution, good drug loading efficiency, and good stability within 21 days. Compared with STS injection, STS-Nano-RBCs extended the drug release time in vitro and in vivo with better repairing effect on oxidative stress-impaired endothelial cells. These results suggest that the nano erythrocyte ghosts system could be used to deliver STS.

## Background

To date, numerous nanoparticles (NPs) delivery has been used to deliver drugs, including polymeric NPs, liposome, microspheres, cell and cell derivatives [1]. As potential drug delivery systems, cell and cell derivatives have been widely researched recently, such as erythrocyte (RBC), tumor cell, stem cell, macrophage, dendritic cell. [2–4]. Among them, erythrocyte widely attracted the attention of researchers. Comparing with other cell drug carriers, erythrocyte is easier to be separated, with better biological compatibility as well as the inherent biological degradation. Besides that, it also has the remarkable long life span of about 3 months in the body [5]. Therefore, small-molecule drugs (doxorubicin [6], dexamethasone sodium phosphate [7]), enzyme (pegademase, adenosine deaminase [8], L-Asprraginase [9]), nucleoside (FdUMP [10], antisense oligodeoxynucleotides [11]), and nanoparticles [12] have been encapsulated in erythrocyte. In recent years, nanoparticles derived from erythrocyte (Nano-RBCs), have strongly attracted the attention of investigators due to the virtue inherited from their parent cells. Besides that, nanoparticles contain therapeutic molecule which could camouflaged themselves with erythrocyte membrane to reduce toxicity [13]. Researches revealed that polymeric nanoparticles loaded in Nano-RBCs showed longer circulation time in blood than PEG-medicated nanoparticles [14]. Besides, coating with Nano-RBC membrane could protect the original nanoparticle from being uptake by macrophage [15]. 1–1.5 × 10^9^ erythrocyte ghosts can split into 1.6 × 10^12^ nanoparticles without considering the loss of erythrocyte membrane. Nanoparticles derived from erythrocyte have high surface area-volume ratio, and its diameter can be small to 100 nm, which helps them easily transmit in vivo [16]. The popular method of Nano-RBC preparation is using hypotonic medium to make erythrocyte change to erythrocyte ghost and then split to smaller vesicles with ultrasonic bath, finally extrude the vesicles through polycarbonate membrane from different pore sizes to unified size gradually [14, 15]. Besides, tip sonicator could also split the Nano-RBCs to around 100 nm directly [16]. So far, a variety of nanoparticles [14, 15] and a small-molecule weight drug (Fasudil, paclitaxel, and doxorubicin) [17, 18] have been encapsulated into Nano-RBC, such as vaccine encapsulated in polymeric nanoparticles and coated with Nano-RBC was developed to prevent the melanoma [19]. Besides, the Nano-RBC with gold nanocages was prepared to improve the efficiency of the photothermal therapy [20].

**Fig. 1:**
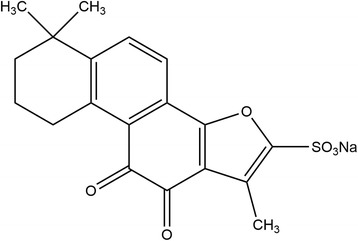
Molecular structure of Sodium Tanshinone IIA sulfonate (STS)

Sodium tanshinone IIA sulfonate (STS, Fig. 1) has been widely used in the treatment of cardiovascular diseases with the effect of arteries expansion and blood flow speedup [21]. Several studies also indicated that STS had not only with pharmacological effect of anti-inflammatory and anti-oxidant in the recovery of tissue ischemia injury [22], but also with the effect of protecting damaged vascular endothelial cells by promoting expression of vascular endothelial growth factor (VEGF) [23]. However, STS eliminates very fast in vivo. The half-life time of STS injected intravenously is less than 0.9 h and 95% drug eliminate in blood within 4 h [24, 25]. To overcome this, numerous dosage forms are applied to achieve longer systemic circulation and better efficacy [24].

In present study, we developed a novel STS-loaded nano-erythrocyte system which was extracted from Sprague dowley (SD) rats. The influence factors of preparation process were investigated to optimize the manufacture process. The stability was tested with the index of particle size and loading efficiency. In vitro and in vivo release behaviors were analyzed to predict its pharmacological effect. Since STS has a protecting function against the oxidative stress induced injury [25], an oxidative stress-impaired model was established with EA.hy926 endothelial cells to assess the repair efficiency of STS-Nano-RBCs and STS injection.

## Methods

### Materials

STS, heparin sodium, 30% hydrogen peroxide solution (H_2_O_2_, 30 wt.% in water), ethyl p-hydroxybenzoate, acetonitrile, and DMSO were made by Aladdin (Shanghai, China). Red blood cell lysis buffer, kaumas blue, cell lysis buffer for western and IP, Nitric Oxide Assay Kit, Total Superoxide Dismutase Assay Kit with WST-8, Lipid Peroxidation MDA Assay Kit, and Reactive Oxygen Species Assay Kit were purchased from Beyotime Biotechnology (Shanghai, China). Amicon Ultra-4 Centrifugal Filter (30 kDa) and Nuclepore Track-Etched Membrane were purchased from Millipore (Boston, USA). 0.5% Triton X-100, 3-(4,5-dimethylthiazol-2yl) -2,5-diphenyltetrazolium bromide (MTT), 1,1-Dioctadecyl −3,3,3,3-tetramethylindodicarbocyanine (DiD), were purchased from Keygen Biotech (Nanjing, China). FITC-dextran (70 kDa) was purchased from TdB Consultancy (Ferndown, UK). Dialysis cassettes (3500 Da) were purchased from Viskase Companies (Houston, USA). 1 × PBS and 10 × PBS (pH 7.4) were purchased from HyClone (USA), fetal bovine serum (FBS), penicillin (10 kU/ml), streptomycin (10 μg/ml) (Penicillin-Streptomycin Solution, 100×), 0.25% Trypsin-EDTA (1×), and Gibco Dulbecco’s Modified Eagle’s Medium (DMEM) were obtained from Invitrogen (USA). The dialysis tube (width is 10 mm, molecular cut-off is 3500 Da) was purchased from Viskase (USA).

### Animals and Ethics

The Sprague Dawley rats, 8 weeks of age, were purchased from the Experimental Animal Center of Southern Medical University of China (SCXK (Guangdong) 2012–0015) and were raised under specific pathogen-free (SPF) condition in the Animal Center of Guangdong Pharmaceutical University. The animal experiments involved in present study were consistent with the guidelines set by the National Institutes of Health and were approved by the Guangdong Experimental Animal Ethics Committee.

### Cells

The human umbilical vein cell line, EA.hy926, was established by fusing primary human umbilical vein cells with a thioguanine-resistant clone of A549 by exposure to polyethylene glycol (PEG). The EA.hy926 cells used in present study were donated by professor Li Ming of the Foundation College of Guangdong Pharmaceutical University.

### HPLC Analysis Method

HPLC method was established by our lab and was used for quantitative determination of STS. The experiment was performed with a Shiseido-ODS C18 column (4.6 × 150 mm); the mobile phase was consisted of methanol and phosphate buffer (pH 3.5) (60:40 v/v); the flow rate was 1 ml/min, and the UV detected wavelength was 271 nm. The temperature of column heater was maintained at 40 °C, and 20 μl sample solution was injected for analysis. The limit of detection (LOD) of STS by HPLC method was 0.02 μg/ml, and the limit of quantitation (LOQ) was 0.06 μg/ml.

### Preparation of STS-Nano-RBCs

STS-Nano-RBCs were prepared with the method reported previously [14]. To prepare erythrocyte ghosts, firstly, blood collected from SD rat put into tube with heparin sodium. Then centrifuged to discard serum and buffy coat, washed RBC three times with 1 × PBS (pH 7.4) carefully to obtain packed RBC. After that, 1 volume packed RBC was incubated with 4 volumes hypotonic 0.1 × PBS at 4 °C for 15 min to obtain erythrocyte ghosts. The erythrocyte ghosts were centrifuged at 2000 rpm for 10 min and washed 3 times with 0.1 × PBS until the supernatant was colorless.

To prepare STS-Nano-RBCs, 1 volume STS (dissolved in 0.1 × PBS) is added into the erythrocyte ghosts and is incubated at 4 °C for 1 h with constant shaking by table concentrator to load STS. Subsequently, 10 × PBS was added to reseal the erythrocyte ghosts at 4 °C for 1 h. The resealed erythrocyte ghosts were washed three times and were resuspended in 1 × PBS. The particle size of drug loaded erythrocyte ghosts was reduced with extrusion method. The erythrocyte ghosts contained STS were extruded 21 cycles by the extruder (ATS engineering Canada) with different size of Nuclepore Track-Etched Membrane (800, 400, and 200 nm). The free STS was removed by ultrafiltration using Amicon Ultra-4 Centrifugal Filter at 5000 rpm for 30 min and was washed with 1 × PBS to obtain the final STS-Nano-RBC system.

In order to screen the optimal drug loading conditions, different influence factors were investigated including the STS concentration (0.2–2.5 mg/ml), loading temperature (4 and 37 °C), loading volume ratio (1:2–3:1), osmotic pressure (0.1 × PBS and 0.3 × PBS) as well as STS loading time.

### Characterization of STS-Nano-RBCs

#### Morphology

To test whether the erythrocyte external aqueous phase could enter into the internal phase when erythrocyte ghosts sealing in hyperosmotic solution, two different fluorescent dyes were used for observation with fluorescence microscope (Carl Zeiss Jena, Germany). Red fluorescent dye DiD (10 μg/ml) and green fluorescent dye FITC-dextran (70 kDa, 100 μg/ml) were incubated with erythrocyte ghosts for 30 min to stain the membrane and cytoplasm, respectively. The structure of STS-Nano-RBCs was examined with a transmission electron microscope. A drop of the sample solution was deposited onto a glow-discharged carbon-coated grid. 5 min later, the grid was rinsed with 10 drops of distilled water. A drop of 1% uranyl acetate stain was added to the grid. The grid was subsequently dried and visualized under a HITACHI H-7650 microscope.

#### Particle Size and Zeta Potential

Beckman Coulter (LS13320, USA) was employed to measure the particle size, size distribution, and surface zeta-potential of STS-Nano-RBCs. The test sample was prepared by diluting STS-Nano-RBCs with 1 × PBS into proper concentration, and the tests were performed in triplicate at room temperature.

#### Protein Content

To investigate the content of membrane protein in erythrocyte and the STS-Nano-RBCs, red blood cell lysis buffer was used to lyses the test samples and was extracted the total protein. SDS-PAGE method was used to analyze the type of proteins. In short, erythrocyte and STS-Nano-RBCs were prepared in SDS sample buffer. The samples were then ran on a polyacrylamide gel electrophoresis apparatus (BioRad, USA) at 100 V for 2 h. Finally, the gel was stained with kaumas blue for 1 h then was visualized using electrophoretic imaging system (Aplegen Omega Lum G, USA).

#### STS Loading Efficiency Assay

Drug concentration of STS-Nano-RBCs system was calculated with HPLC method. 50 μl STS-loaded nano-RBCs were added into 3950 μl RBC lysis buffer. The test samples were vortexed for 3 min, collected the lysis solution 200 μl, added 2 ml methanol to extract STS, and then centrifuged at 10000 rpm for 10 min, and 20 μl supernatant was taken to analyze the STS content with HPLC method (Hitachi Chromaster with 5110 pump, 5210 Autosampler, 5310 Column Oven, 5410 UV detector) described above. Drug loading efficiency (LE%) was measured with Eq. 1.

1 $$ \mathrm{L}\mathrm{E}\% = {M}_{\mathrm{in}}/{M}_{\mathrm{total}}\times 100\% $$

Where, *M*_in_ = the entrapment amount of STS in STS-Nano-RBCs, *M*_total_ = the total amount of STS in STS-Nano-RBCs.

### Stability

The centrifugal stability of STS-Nano-RBCs was assessed by centrifuging samples 10 min with different speed (2000–12000 rpm). The turbulence stability of STS-Nano-RBCs was also evaluated. In short, STS-Nano-RBC was passed through a 4.5-gauge needle with the flow rate of 10 ml/min, which was comparable with the blood flow rate in vivo. The number of passes was varied (10–30 times). Next, 10 K MWCO Amicon Ultra-4 Centrifugal Filters were used to isolate free STS for HPLC analysis. STS leakage rate was used as the index to evaluate the system stability. Samples were stored at 4 °C for 21 days to investigate its storage stability, and at given time point, indexes including appearance, drug leakage rate, particle size, and zeta potential were used to evaluate the short-term stability of STS-Nano-RBCs.

### In Vitro Release

The drug release behavior of STS-Nano-RBCs system was evaluated with dialysis method in PBS (pH7.4) at 37 °C. 1 ml concentrated STS-Nano-RBCs was placed into dialysis tube and immersed in 250 ml PBS. The dialysis tube is 10 mm wide, and the molecular cut-off is 3500 Da. At each given time point, 1 ml released solution were collected and were replenished with equal volume of PBS solution. For comparison, the release of STS injection was also tested. All collected solution was analyzed with HPLC method described before.

### In Vivo Pharmacokinetics and Biodistribution Study

Pharmacokinetic characteristics of STS-Nano-RBCs were conducted in adult SD rats. 12 SD rats were divided into 2 groups, and test samples were intravenously injected through rat tail vein with the dose of 5 mg/kg; the concentration of STS-Nano-RBC and STS was 1.1 mg/ml. At each time points, the blood was collected and centrifuged (2000 rpm for 10 min) to separate the plasma. After that, 50 μl ethyl p-hydroxybenzoate (1 μg/ml) and 1 ml acetonitrile were added into plasma to extract STS. The mixture was vortex-mixed for 1 min followed by centrifugation at 10000 rpm for 10 min. The supernatant was evaporated under nitrogen atmosphere condition and was redissolved with 200 μl mobile phase before HPLC assay. The pharmacokinetic parameters were calculated from the plasma concentrations of STS with DAS2.0 software system. The pharmacokinetic parameters estimated were maximum plasma concentration (*C*_max_), half-life (*T*_1/2_), the area under the plasma concentration-time curve (AUC), and the mean residence time (MRT).

Next, we investigated the tissue distribution of STS-Nano-RBC in SD rats to further evaluate its potential as a delivery vehicle. We performed the biodistribution study on 8 week-old SD rats (190–210 g, *n* = 12 per group, half male, half female). SD rats were injected STS-Nano-RBC or STS via tail vein, each rat was injected with approximately 1.0 mg of STS injection or STS-Nano-RBC. At each time point (12, 24, and 36 h) post-injection of drug, blood was collected from the rat eye vein plexus, four rats of each group were euthanized, and their organs (hearts, livers, spleen, lungs, kidney) were extracted and weighed. STS content in each group was measured with HPLC method described before.

### Cell Viability

MTT assay was conducted to identify the biocompatibility of STS-Nano-RBCs. EA.hy926 cell was seeded at the density of 10000 cells per well in 96-well plate and with 100 μl DMEM. When 80% cells confluence was observed, the growth media were replaced with equal volume of test samples (STS, Nano-RBCs and STS-Nano-RBCs, all dissolved in DMEM) with various concentrations for 24 h. Then, the media were refreshed and were incubated with 20 μl MTT (5 mg/ml in PBS) for another 4 h. Finally, the culture medium was removed, 100 μl of DMSO was added, and the absorbance was measured at 570 nm with a microplate reader (BioRad, USA).

### Protective Effect on Oxidative Stress-Impaired Cell

In order to study the repairing effect of STS-Nano-RBC on oxidative stress impairs EA.hy926 cell, we adopted the method published before [26]. In short, an oxidative stress-impaired model was established with 750 μM H_2_O_2_ to evaluate the protective and to repair effect of STS-Nano-RBCs on oxidative stress-impaired cell. For nitric oxide (NO), superoxide dismutase (SOD) and MDA assay, EA.hy926 cells were seeded in 96-well plate with suitable density. When 80% cell confluence was observed, the growth media was replaced with 100 μl H_2_O_2_ (750 μM) and was incubated for 12 h. Then the media was replaced with various concentration of STS-Nano-RBC. 12 h later, the supernatant (or cell lysis solution) was collected and analyzed with Nitric Oxide Assay Kit, Total Superoxide Dismutase Assay Kit, and Lipid Peroxidation MDA Assay Kit. The repair efficiency of STS-Nano-RBCs was also observed with fluorescence microscope.

### Statistical Analysis

All experimental data in this study were shown as mean ± standard deviation (SD). Statistical comparisons were calculated by using Student’s *t* test, *p* values of <0.05 was taken to indicate statistical difference.

## Results and Discussion

### Preparation and Characterization of STS-Nano-RBCs

**Fig. 2:**
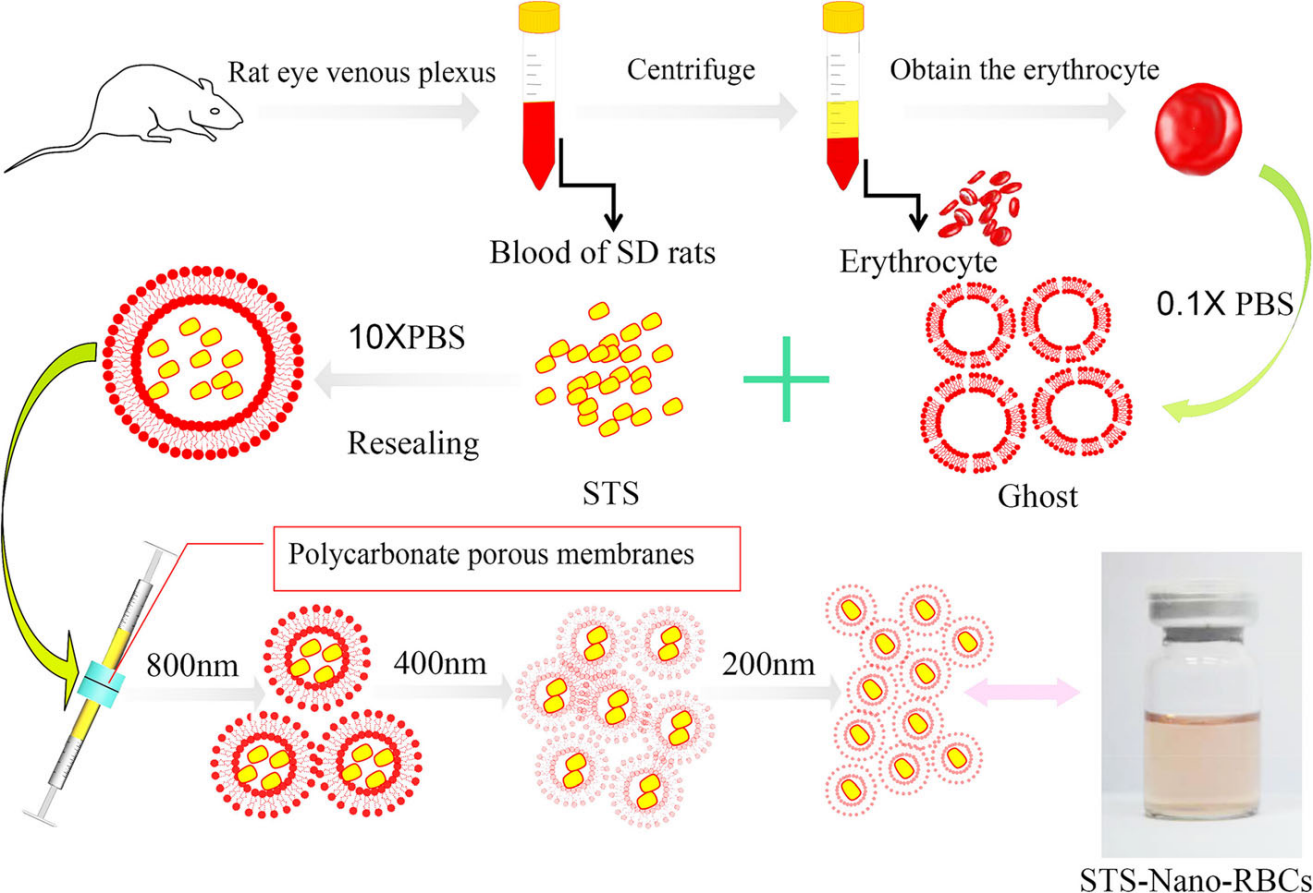
Schematic illustration of STS-Nano-RBCs preparation process

Nowadays, nanoparticle derived from erythrocyte has widely attracted researchers’ attention due to its strong biocompatibility and good inherent biodegradation in vivo. In present study, we developed a new drug carrier system using nano-size erythrocyte ghosts to load water-soluble drug STS for delivery. STS-Nano-RBCs were prepared through three major steps (shown in Fig. 2): preparing the erythrocyte ghost, loading STS into ghosts, and reducing its particle size into nano scale. It is worth noting that rat hemoglobin in 4 °C is not soluble and undergoes gelling and precipitation, which has not happened when human or other mammal RBCs were used; the data in present study was unique for rat RBCs.

**Fig. 3:**
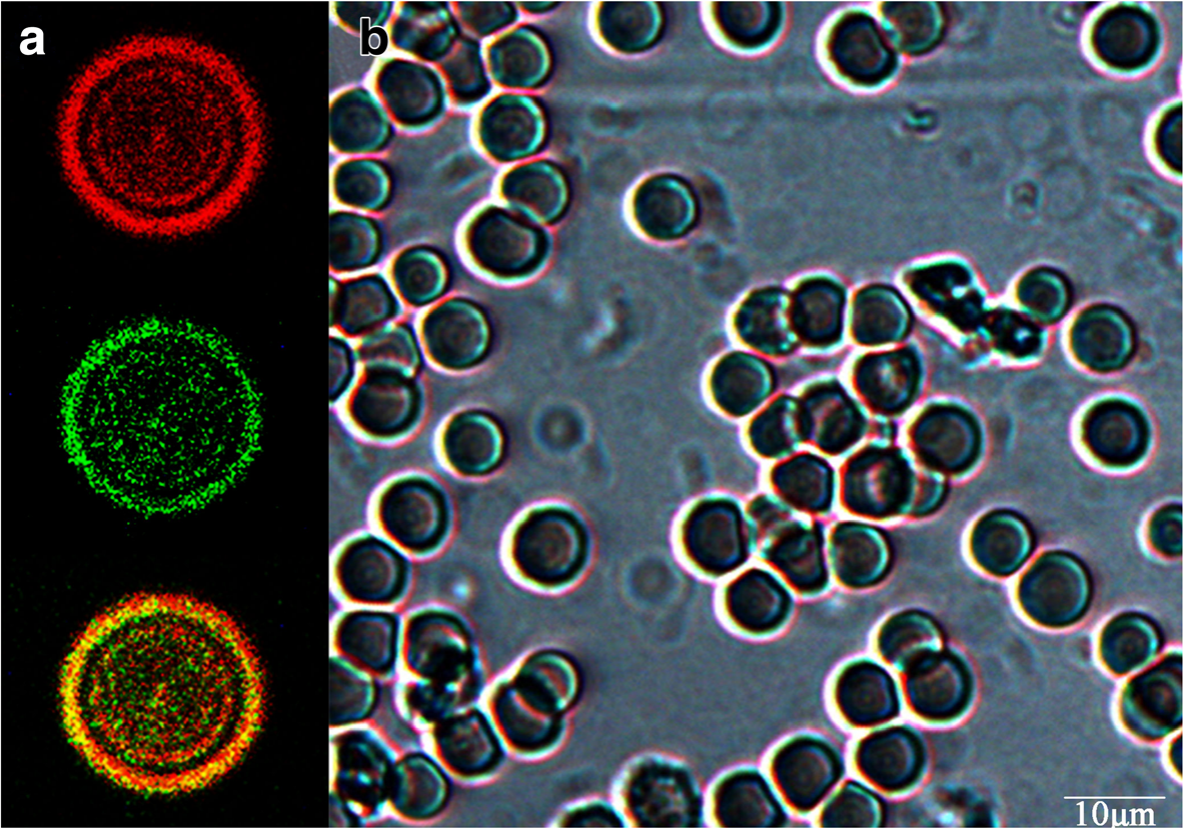
Fluorescent images of erythrocyte ghosts stained with the DiD (*red*) and FITC-dextran (*green*) and merged image (**a**); in *bright field* (**b**)

In hypotonic conditions, the small pores of erythrocytes membrane will open, which result in the entry of external aqueous phase and dissolved materials. This phenomenon could be observed in Fig. 3. As can be seen, the erythrocytes membrane could be stained by DiD (red), and FITC-dextran (green) solution, which indicated that STS, as a hydrophilic small-molecule API, could be encapsulated into erythrocytes too, which was consistent with previous reports [17, 18].

**Fig. 4:**
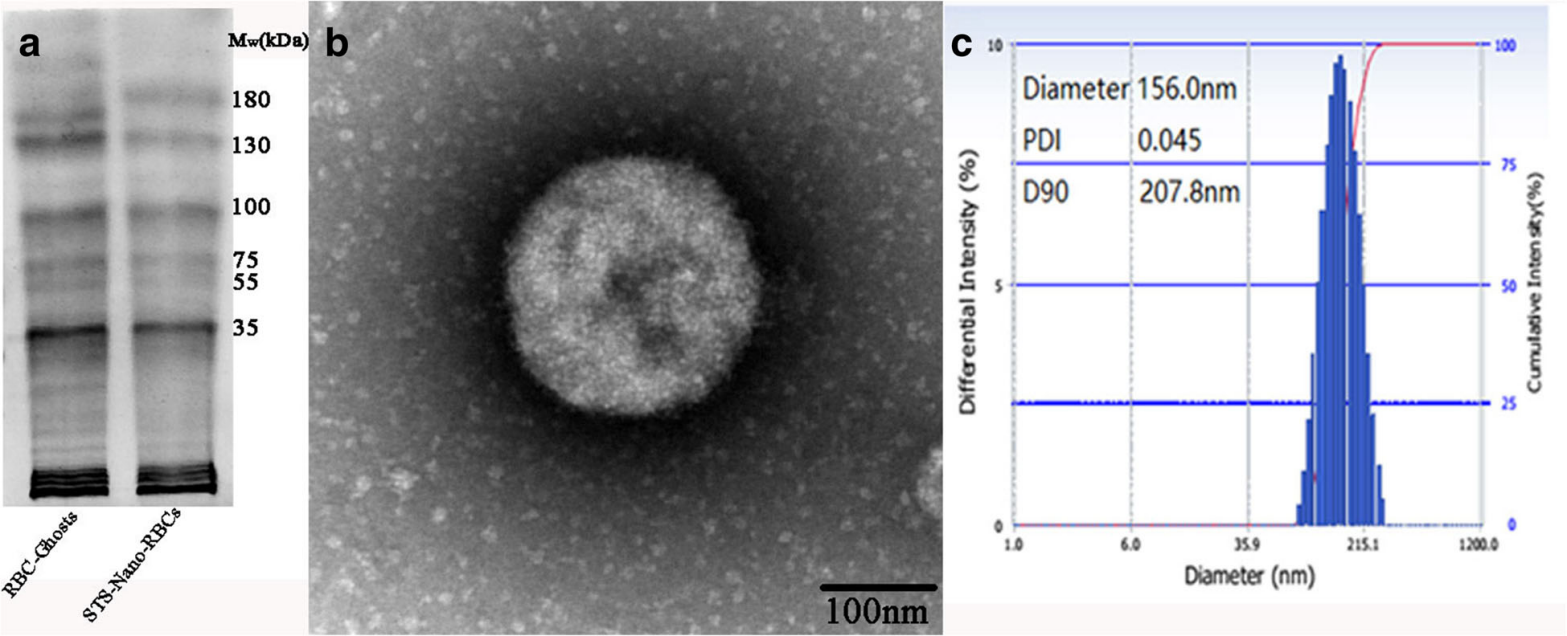
**a** Membrane proteins retention of erythrocyte ghosts and STS-Nano-RBCs. **b** TEM image of STS-Nano-RBCs. **c** The particle size distribution of STS-Nano-RBCs

The particle size distribution of final STS-Nano-RBCs was measured with DLS method and then visualized using transmission electron microscopy. As shown in Fig. 4, nano particles were spherical, and the average diameter of STS-Nano-RBCs was around 156 nm with PDI of 0.045, which indicated a relatively narrow and a unified size distribution. The zeta-potential, caused by negatively charged proteins on the membrane of STS-Nano-RBCs was −2.34 mv, and it was helpful to keep the system stable due to electrostatic forces.

One of the most important advantages to use erythrocytes as a drug delivery system is that the functional components contain immunosuppressive proteins on erythrocyte membranes, which could inhibit macrophage uptake and therefore prolonged the circulation time [27]. As shown in Fig. 4, SDS-PAGE analysis result revealed that in both samples, erythrocyte ghosts contain all major protein fractions: α-spectrin, β-spectrin, actin, glyceraldehyde-6-phosphate dehydrogenase, stomatin-tropomyosin, and peroxiredoxin and reduced globin chains. However, the result also revealed that several major bands of protein color in Nano-RBC group was weaker than in RBC group, which means the preparation process might not cause the loss of protein species, but could cause the loss of some protein content.

**Fig. 5:**
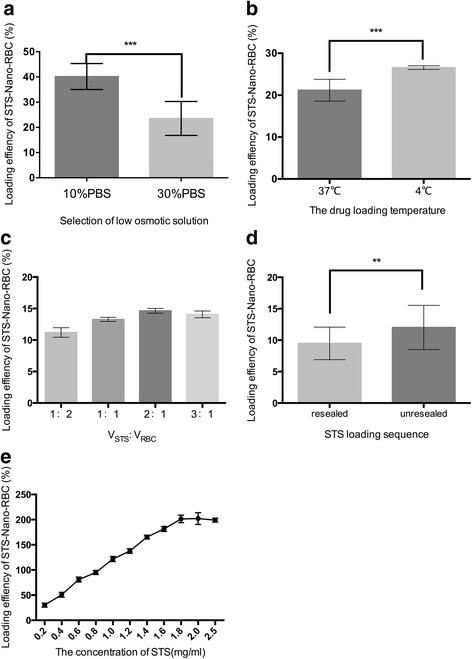
Optimization of STS loading. **a** Effect of osmotic pressure. **b** Effect of temperature. **c** Effect of volume ratio of STS to erythrocyte. **d** STS loading before and after erythrocyte ghosts resealing. **e** Effect of STS concentration. The data are presented as mean ± SEM (*n* = 3), *, **, *** indicate *p* < 0.05, <0.01, and <0.001, respectively

To optimize the STS-Nano-RBCs preparation process, several influences were investigated in this study. As shown in Fig. 5, lower hypotonic solution (0.1 × PBS) could effectively load STS into erythrocyte ghosts, and the drug loading efficiency was 39.7%, which is higher than that obtained in 0.3 × PBS. This result was consistent with previous study, which proved that decreased osmolarity could result in the creation of more pores in the membranes, allowing the entry of water and dissolved materials [17]. Temperature is also an important parameter that could influence drug loading efficiency. As can be seen in Fig. 5, STS loading rate at 4 °C was higher than 37 °C, which might be caused by mobility of membrane phospholipids and long time pores open in low temperature. It is worth noting that STS loading rate was increased with the increase of STS concentration, which was ranged from 0.2 to 1.8 mg/ml and reached to equilibrium when STS concentration exceeded 2.0 mg/ml. The optimal volume ratio of STS solution to erythrocyte ghosts was 2:1, and higher loading efficiency could be obtained when STS was added before erythrocyte ghosts resealing.

### Stability of STS-Nano-RBCs

**Fig. 6:**
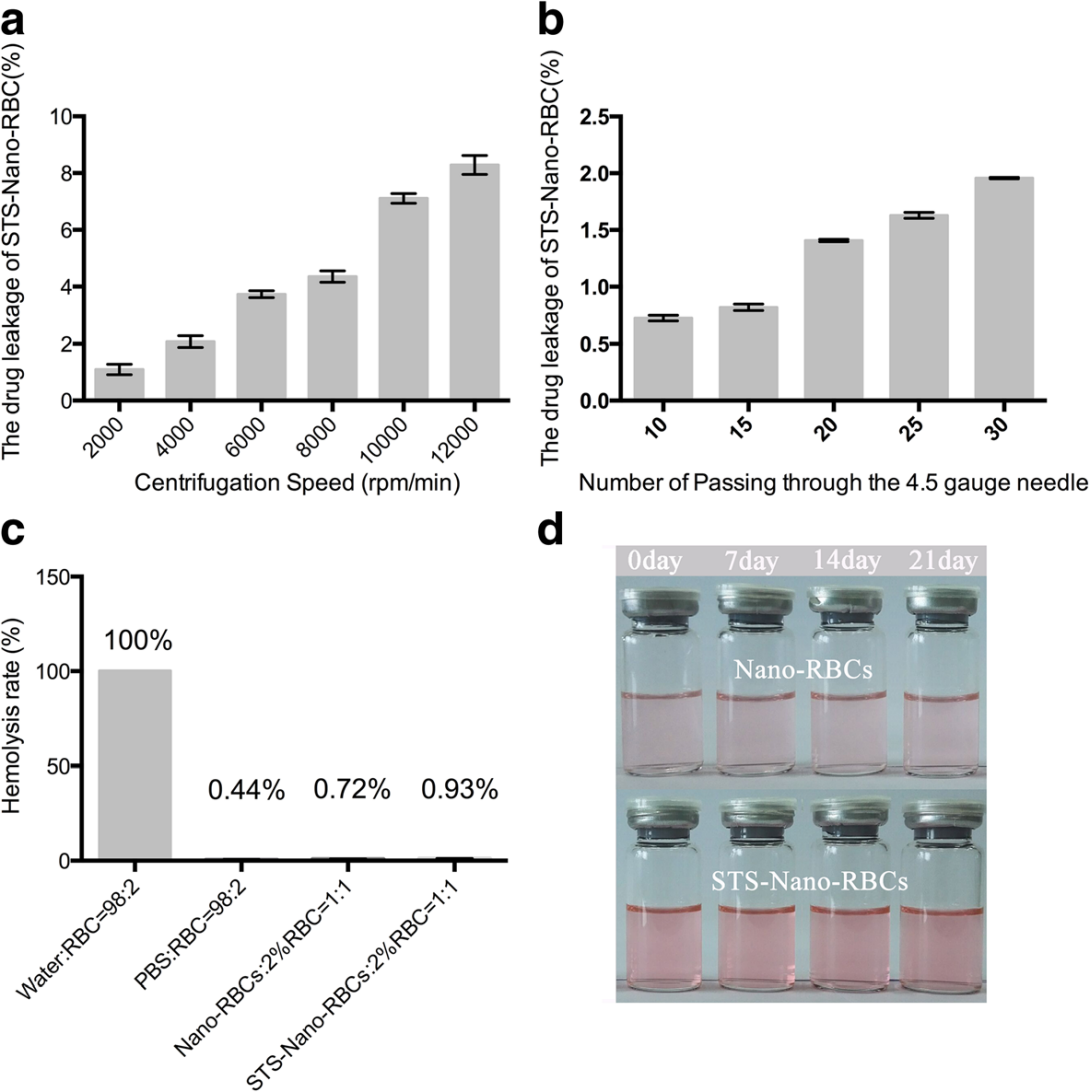
In vitro stability of STS-Nano-RBCs. **a** Centrifugation stability. **b** Turbulence stability. **c** Hemolysis test. **d** The stability of Nano-RBCs and STS-Nano-RBCs in PBS at 4 °C within 21 days. The data are presented as mean ± SEM (*n* = 3)

Formulation stability was of great importance for the preparation and application of nano carrier systems. In the present study, centrifugation and turbulence stability were used to assess the stability of STS-Nano-RBCs. The result, as shown in Fig. 6a, revealed that STS leakage rate was increased with centrifugation speed. When the centrifugation speed was 10000 rpm, 7.1% of STS was moved from internal to external water phase. For turbulence stability, the result was shown in Fig. 6b, when the number of passes increased to 30 times, the leakage rate of STS was just 1.96%, which indicated that STS-Nano-RBCs could withstand the impact of blood flow in vivo. When STS-Nano-RBCs were administrated intravenously, it could be diluted and interacted with blood cells. The formulations should keep stable with high dilution ratio and could not cause hemolytic reaction. Here, the potential interaction of STS-Nano-RBCs with the blood cells was measured using hemolysis index. The data showed that compared with 100% hemolysis caused by water, STS-Nano-RBCs caused minimal hemolysis (As shown in Fig. 6c). The long-term stability of STS-Nano-RBCs was also investigated by testing the indexes including appearance, particle size, zeta potential, and entrapment efficiency within 21 days, as shown in Fig. 6d, little particles aggregation was found. The average diameter of STS-Nano-RBCs was increased slightly from 160.2 to 174.4 nm, and the surface potential could maintain negatively charged (Table 1). STS entrapment efficiency was about 40% during test period, which proved that STS-Nano-RBC has good storage stability.

**Table 1:** The parameters of storage stability of STS-Nano-RBCs

Time (day)	Drug loading efficiency (%)	Zeta potential (mv)	Size (nm)	Polydispersity index
0	40.1	−2.17	160.2 ± 4.5	0.047
7	39.8	−2.18	163.2 ± 5.7	0.047
14	39.6	−2.22	166.9 ± 3.3	0.051
21	39.5	−2.23	174.4 ± 3.4	0.056

The data are presented as mean ± SEM (*n* = 3)

### In Vitro and In Vivo Drug Release and In Vivo Biodistribution

**Fig. 7:**
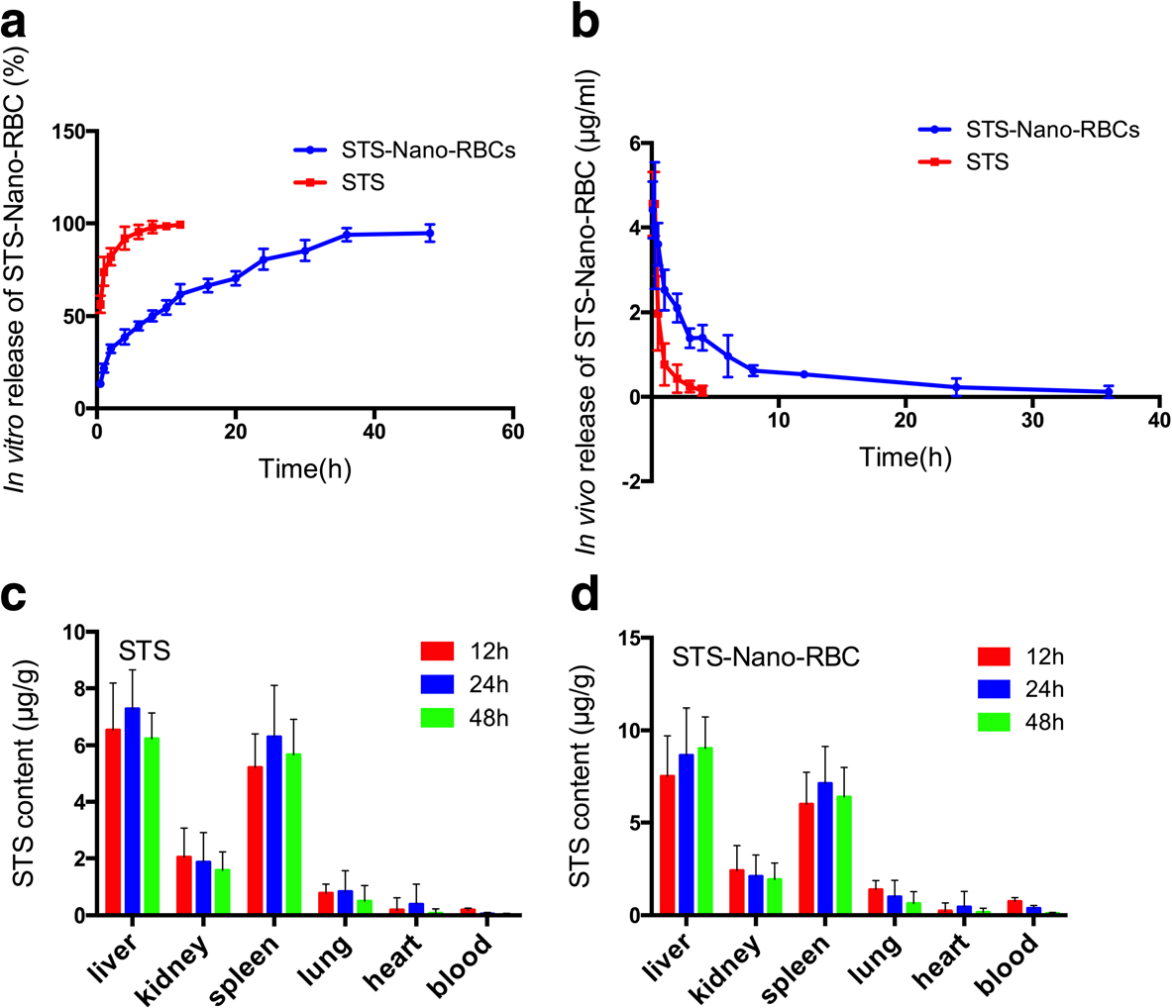
In vitro release profile of STS-Nano-RBCs in PBS solution at 37 °C (**a**). **b** In vivo release profile of STS-Nano-RBCs in rats. **c** Biodistribution of STS-Nano-RBC and STS injection. After intravenously injecting STS-Nano-RBC and STS into SD rats, the organs at each time point were collected (12, 24, 48 h respectively), were homogenized, and were quantified for STS content. The data is presented as mean ± SEM (*n* = 6)

To assess the release characteristic, dialysis method was used to investigate the STS release from STS-Nano-RBCs in PBS solution at 37 °C. STS-Nano-RBCs group showed sustainable release compared with STS injection. As shown in Fig. 7a, STS injection presents typical fast drug release behavior, within 6 h almost 97% STS could be tested outside the dialysis tube. On the contrary, STS-loaded Nano-RBCs showed sustained drug release property in 48 h with the mean release rate of 1.98% per hour, which was much slower than STS injection (16.67% per hour).

When two formulations were administrated into SD rats, as shown in Fig. 7b and Table 2, the result indicated that compared with STS injection, using Nano-RBCs as drug delivery system could prolong the drug release time. In addition, *C*_max_ of STS-Nano-RBC group and STS injection group were 4.053 and 4.386 μg/ml, respectively. Compared with STS injection group, half-life (t1/2) of STS-Nano-RBC group increased from 2.59 to 6.92 h, and the mean residence time (MRT) increased from 2.23 to 8.14 h. Drug concentration could be detected after 36 h in STS-Nano-RBCs group, and the area under the drug concentration-time curve was 19.69 μg/ml · h, which indicated that STS-Nano-RBCs could achieve sustained release. The release equation of STS-Nano-RBC group was shown as Eq. 2, and the model followed by STS-Nano-RBC was two-compartment model.

**Table 2:** Pharmacokinetics parameters calculated by DAS2.0 software

	*C*_max_ (μg/ml)	*T*_1/2_ (h)	AUC_0−∞_ (μg/ml · h)	MRT (h)
STS	4.386	2.591	3.394	2.232
STS-Nano-RBC	4.053	6.917	19.698	8.141

*C*_*max*_ maximum plasma concentration (μg/ml), *T*_*1/2*_ half-life of STS in plasma (h), *AUC*_*0−∞*_ area under the curve (μg/ml · h), *MRT* mean retention time (h)

2 $$ \mathrm{C}={\mathrm{Ae}}^{\hbox{-} {\upalpha}_{\mathrm{t}}} + {\mathrm{Be}}^{\hbox{-} {\upbeta}_{\mathrm{t}}} $$

Where A = 2.423, B = 1.687, α = 0.856, β = 0.093.

Subsequently, we used SD rats to conduct the in vivo biodistribution study. Figure 7c, d showed the STS content per gram of tissue, at 12 h after the injection, STS-Nano-RBC showed hepatic and splenic uptake of 7.5 and 6.1 μg/g tissue, respectively, as compared to 6.4 and 5.2 μg/g tissue by STS injection. After accounting for the tissue mass, it can be observed that with the injection time extended, STS-Nano-RBC observed in the liver and spleen was increased, while in blood was decreased. Liver and spleen, as the two primary organs of RES, contained the highest amount of STS-Nano-RBCs, which also explained the fast blood elimination of STS-Nano-RBC.

It is well known that the cycle time of erythrocyte in rats is about 1 month, compared with autologous RBC, the sustained release effect of STS-Nano-RBC was quite limited. Combined with Fig. 4, it can be speculated that the surface protein of erythrocyte, not only the protein species but also the content, played an important role on its cycle time. The surface glycoproteins of erythrocyte, such as CD47, as the signal molecule, could protect RBCs from damage and elimination from body by inhibiting the phagocytosis of macrophage through binding to SIRPα. Loss or just decrease of surface glycoproteins of erythrocyte, such as CD47, could inhibit the “do not eat me” signal, which will cause STS-Nano-RBC to be phagocytosed by macrophage [28]. Besides, Nano-RBC, as the derivative of RBC, destructed the integrity of mother cell, and the process of drug encapsulation typically require multistep manipulations involving cell isolation, incubations for several times, and washings, which could harm the cell membrane and thus lead to transposition of PS from the inner leaflet of the plasma membrane to the RBC surface, which might accelerate elimination of Nano-RBC in rats [29]. In addition, the effect of polymeric nanoparticles on biocompatibility of carrier erythrocyte has been investigated before; the result indicated that non-covalent adsorption of model NPs (200 nm) to mouse and human RBC could affect their sensitivity to osmotic stress, low level shear stress, vigorous mechanical insult, and agglutination. Although the result showed non-covalent adsorption of model NPs to mouse and human RBC is not detrimental at ratios of and below NP/RBC 200:1, it inspired us to assume that whether Nano-RBC attached on RBC in rats increases the RBC susceptibility to complement-mediated lysis [30].

### Cell Viability Assay

**Fig. 8:**
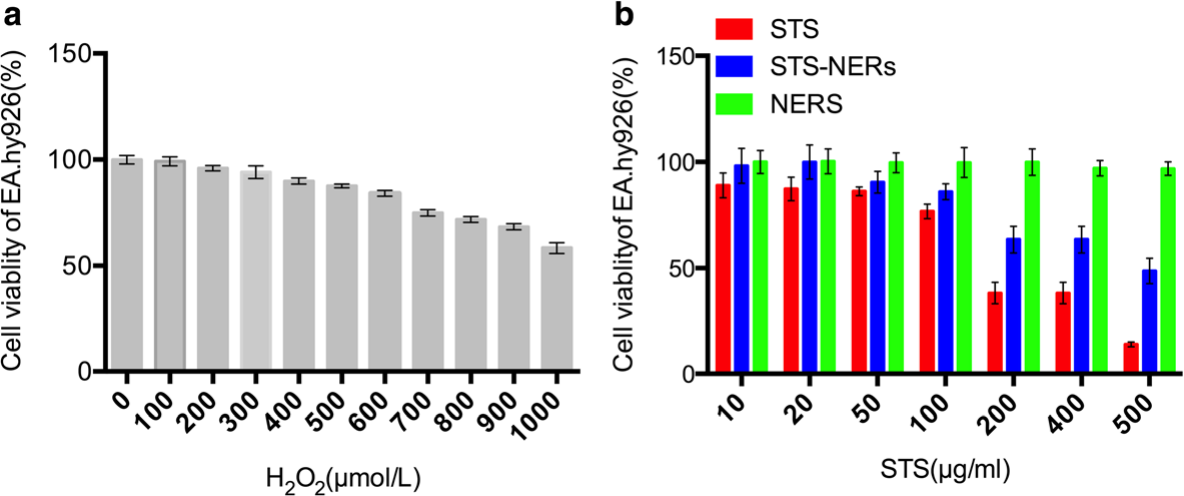
**a** Cell viability of H_2_O_2_ in EA.hy926 cells. **b** Cell viability of STS injection, Nano-RBCs and STS-Nano-RBCs in EA.hy926 cells. The data are presented as mean ± SEM (*n* = 6)

In order to select an appropriate concentration of H_2_O_2_ to build oxidative stress-impaired cell model, incubated cells with different concentration of H_2_O_2_ for 12 h then determined cell vitality with MTT method, as shown in Fig. 8a, when H_2_O_2_ concentration was 700 μM, the cell vitality was 74.77%, when H_2_O_2_ concentration was 800 μM, the cell vitality was 71.51%. In the present study, we choose 750 μM H_2_O_2_ to build oxidative stress-impaired cell model.

Application safety is a prerequisite for drug delivery system, and here, the cell cytotoxicity was tested to evaluate the biocompatibility of STS-Nano-RBCs. EA.hy926 cells were chosen to assess the cell viability of STS-Nano-RBC with MTT method. As shown in Fig. 8b, the concentration of STS-Nano-RBCs was ranged from 10 to 500 μg/ml. The MTT results indicated Nano-RBCs showed little cytotoxicity to EA.hy926 cells. Cell viability of both STS injection group and STS-Nano-RBC group were decreased with the increase of STS concentration. It was worth noting that when the concentration of STS was higher than or equal to 100 μg/ml, STS-Nano-RBCs group showed more cytotoxicity than STS group. This might be due to the difference of STS concentration in cells at a given time. It is well known that the way in which small-molecule weight drug and nanoparticles enter cells is different, which might cause different concentration of STS at a given time and thus result in different cell viability. Since cell survival rate was less than 50% when STS-Nano-RBC concentration was greater than or equal to 200 μg/ml, we selected 10–150 μg/ml as the concentration range to examine the protection of STS against oxidative stress injury cells.

### Repairing Effect of STS-Nano-RBCs on Oxidative Stress Impairs EA.hy926 cell

**Fig. 9:**
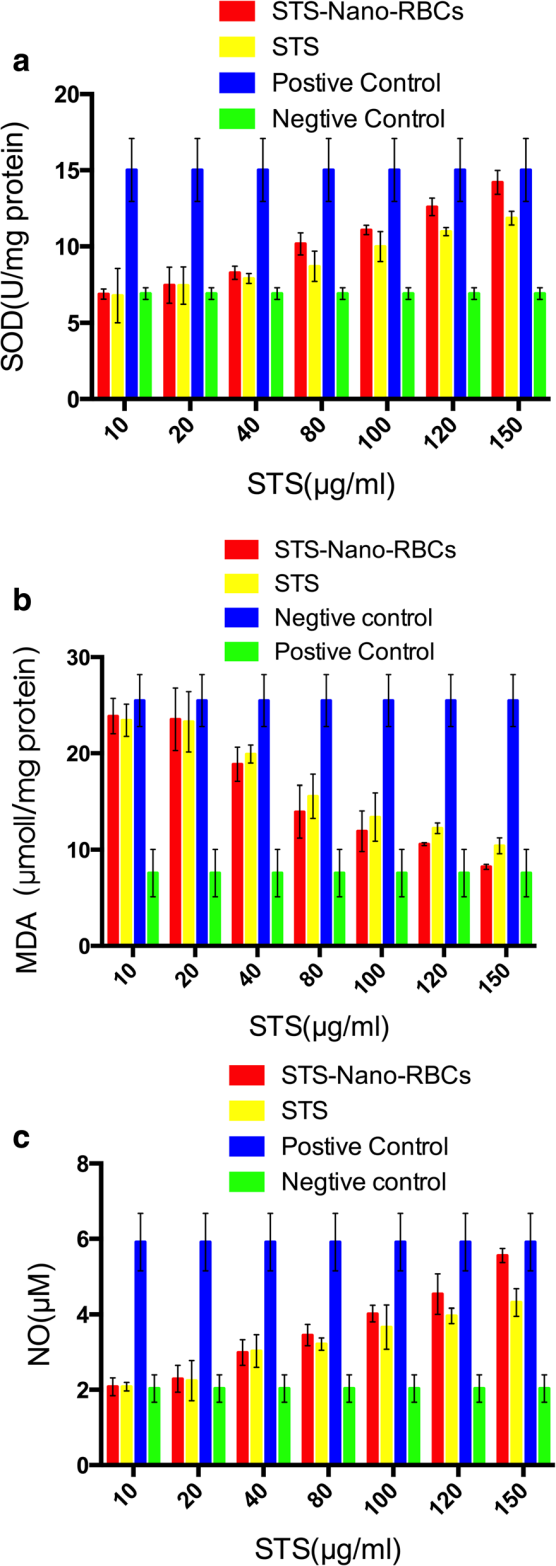
Protective effect of STS-Nano-RBC group and STS injection group on oxidative injured EA.hy926 cells by detecting the content of SOD (**a**), NO (**b**), and MDA (**c**). The data are presented as mean ± SEM (*n* = 6)

**Fig. 10:**
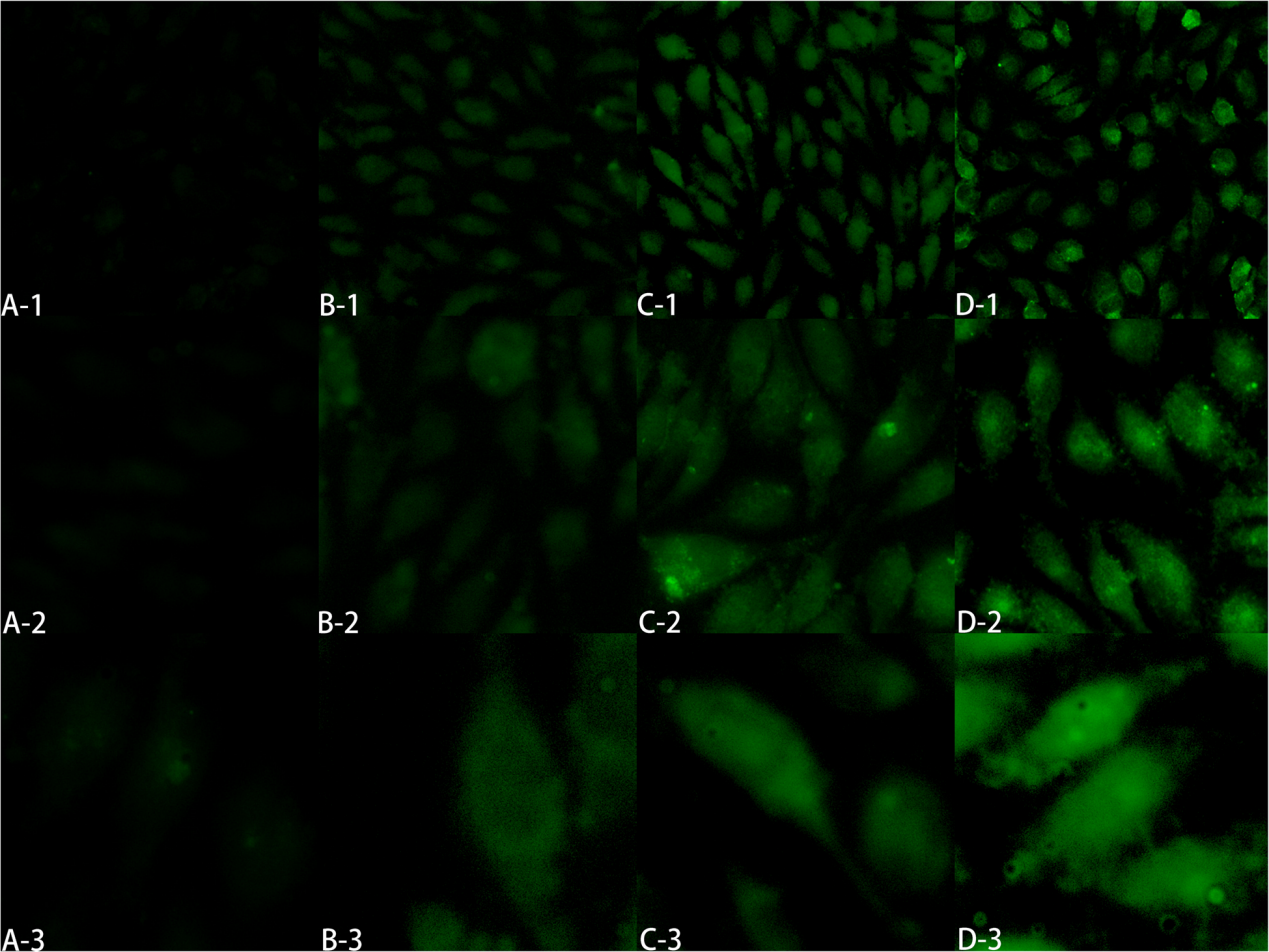
Fluorescent images of oxidative damaged EA.hy926 cells observed using H2DCFDA probe. **a** The positive control group. STS-Nano-RBCs repairing group (**b**); STS repairing group (**c**) and the negative control group (**d**); 1 = 4×, 2 = 10×, 3 = 40×

Reactive oxygen species (ROS), including hydrogen peroxide (H_2_O_2_), hydroxyl (OH^−^), nitric oxide (NO), and oxygen-free radical (O^2−^), are involved in multiple physiological and pathological reactions [31]. Excessive ROS can lead to cell apoptosis, and numerous studies have revealed that ROS played an important role in oxidative stress [32, 33]. Under the stimulation of cell-injured signals and inflammatory mediators, the endothelial cells will produce ROS in the intracellular compartment [34]. Since the redox homeostasis is the key to maintain normal cells physiological activity [35], excessive ROS will be removed from cell by enzymatic or non-enzymatic reactions. Superoxide dismutase (SOD), used to clean out the extra ROS, could maintain internal balance, and its concentration has a positive correlation with ROS level inside the cell [36]. ROS could also cause phospholipids peroxidation of polyunsaturated fatty acids on the cell membrane, and further disorder the uniform structure of the cell membrane. Malondialdehyde (MDA), as the final product of lipid peroxidation, is believed to reflect the phospholipids peroxidation degree [37, 38]. As shown in Fig. 9, the SOD level was raised with the increase of STS concentration. When STS concentration was 80 μg/ml or higher, STS-Nano-RBCs group showed higher SOD level compared with STS injection group. NO, as an effective vasodilator molecule, could improve the blood flow of the ischemia reperfusion and could regulate the recovery of damaged endothelial cells. When STS concentration was 80 μg/ml or higher than 100 μg/ml, higher content of NO could be detected in STS-Nano-RBCs group than that in STS injection group. Similar results could be seen in Fig. 10 that cells treated with STS-Nano-RBCs could remove more ROS and cells therefore presented weak green fluorescence (Fig. 10 (B-1)). However, positive group showed strong green fluorescence, which means ROS level was higher. The result demonstrated that STS-Nano-RBCs showed better capability of repair oxidative stress-impaired endothelial cells. Although STS-Nano-RBC group showed a better repair effect at the concentrations of 80–150 μg/ml, there was no significant difference in the repair effect of the two groups at low concentrations (10–40 μg/ml). This may be due to the inability to reach the repair threshold when the STS concentration is lower than or equal to 40 μg/ml. The STS-Nano-RBC group showed a better repair effect when the STS concentration was or higher than 80 μg/ml, probably because the Nano-RBC changed the way STS entered the cell, which caused different STS concentration in cells, and then showed a better repair effect. In conclusion, further studies on how Nano-RBCs improve STS efficacy require experimental validation.

## Conclusions

In this study, we developed a drug carrier system of nano erythrocyte ghosts to deliver water-soluble drug STS and to investigate its biological characteristics. STS loaded into nano erythrocyte ghosts was found to exert several benefits including good stability, sustained drug releasing behavior, as well as bioavailability improvement. It could also extend in vivo circulation time. Based on the pharmacological experiment, it is evidence that STS-loaded nano erythrocyte ghosts could upgrade the repairing effect of damaged endothelial cells and can serve as a drug delivery system to deliver STS.

## Abbreviations

AUC_0−∞_: Area under the curve
*C*_max_: Maximum plasma concentration
DiD: 1,1-Dioctadecyl −3,3,3,3-tetramethylindodicarbocyanine
MDA: Malondialdehyde
MRT: Mean retention time (h)
MTT: 3-(4,5-dimethylthiazol-2yl)-2,5-diphenyltetrazolium bromide
Nano-RBCs: Nanoparticles derived from erythrocyte
NPs: Numerous nanoparicles
RES: Reticuloendothelial system
ROS: Reactive oxygen species
SOD: Superoxide dismutase
STS: Sodium Tanshinone IIA sulfonate
t_1/2_: Half-life of STS in plasma
VEGF: Vascular endothelial growth factor
